# Restoration of Knee Function After Tibial Tubercle Proximalization for Infrapatellar Contracture Syndrome Following Patellar Fracture Surgery

**DOI:** 10.7759/cureus.89923

**Published:** 2025-08-12

**Authors:** Kazu Matsumoto, Daichi Ishimaru, Kazuki Sohmiya, Nobuo Terabayashi

**Affiliations:** 1 Department of Orthopedics, Gifu Seiryu Hospital, Gifu, JPN

**Keywords:** infrapatellar contracture syndrome, patella baja, patella fracture, range of motion, tibial tubercle proximalization

## Abstract

Infrapatellar contracture syndrome (IPCS) is a rare but functionally disabling condition characterized by anterior knee fibrosis and patellotibial impingement. We report the case of a 59-year-old woman who developed IPCS after undergoing tension band wiring for a patellar fracture, presenting with pain, quadriceps weakness, and marked patella baja (Insall-Salvati ratio: 0.377). Proximalization of the tibial tubercle by 15 mm restored patellar height and improved knee flexion from 120° preoperatively to 150° at the one-year follow-up. At two years, the patient achieved full squatting and floor sitting (seiza), with no extension lag, quadriceps strength of 86.4 N (contralateral: 96.6 N), and an improved Timed Up and Go score from 12.89 to 6.86 seconds. Tibial tubercle proximalization may be an effective tendon-sparing option for IPCS with patella baja, enabling restoration of patellar height, improved range of motion, and early rehabilitation without direct tendon manipulation.

## Introduction

Infrapatellar contracture syndrome (IPCS) is an uncommon but debilitating condition characterized by restricted knee range of motion (ROM) due to arthrofibrosis, soft tissue contracture, and entrapment of the patella following trauma or surgery [1]. Paulos et al. [2] classified IPCS into primary and secondary cases and further divided it into three stages, prodromal, active, and residual, associated with progressive quadriceps atrophy and functional impairment. Patella baja and patellotibial impingement may occur in some chronic cases, leading to anterior knee pain and difficulty performing daily activities such as squatting or stair climbing. Typical risk factors include prior anterior knee surgery or trauma, particularly procedures involving open reduction and internal fixation for patellar fractures [3-5].

Conservative treatment often fails in chronic cases, and surgical options remain controversial. Patellar tendon lengthening or reconstruction techniques, including Z-plasty and graft augmentation, have been described [6,7] but require direct tendon manipulation, increasing the risk of vascular compromise or recurrent fibrosis. Tibial tubercle proximalization has been reported as a tendon-sparing alternative that restores patellar height and relieves impingement [8,9], although evidence in IPCS remains limited.

We report a case of post-traumatic IPCS with patella baja refractory to conservative treatment, successfully managed by tibial tubercle proximalization, resulting in restoration of patellar height, pain reduction, and improved knee function.

## Case presentation

**Figure 1:**
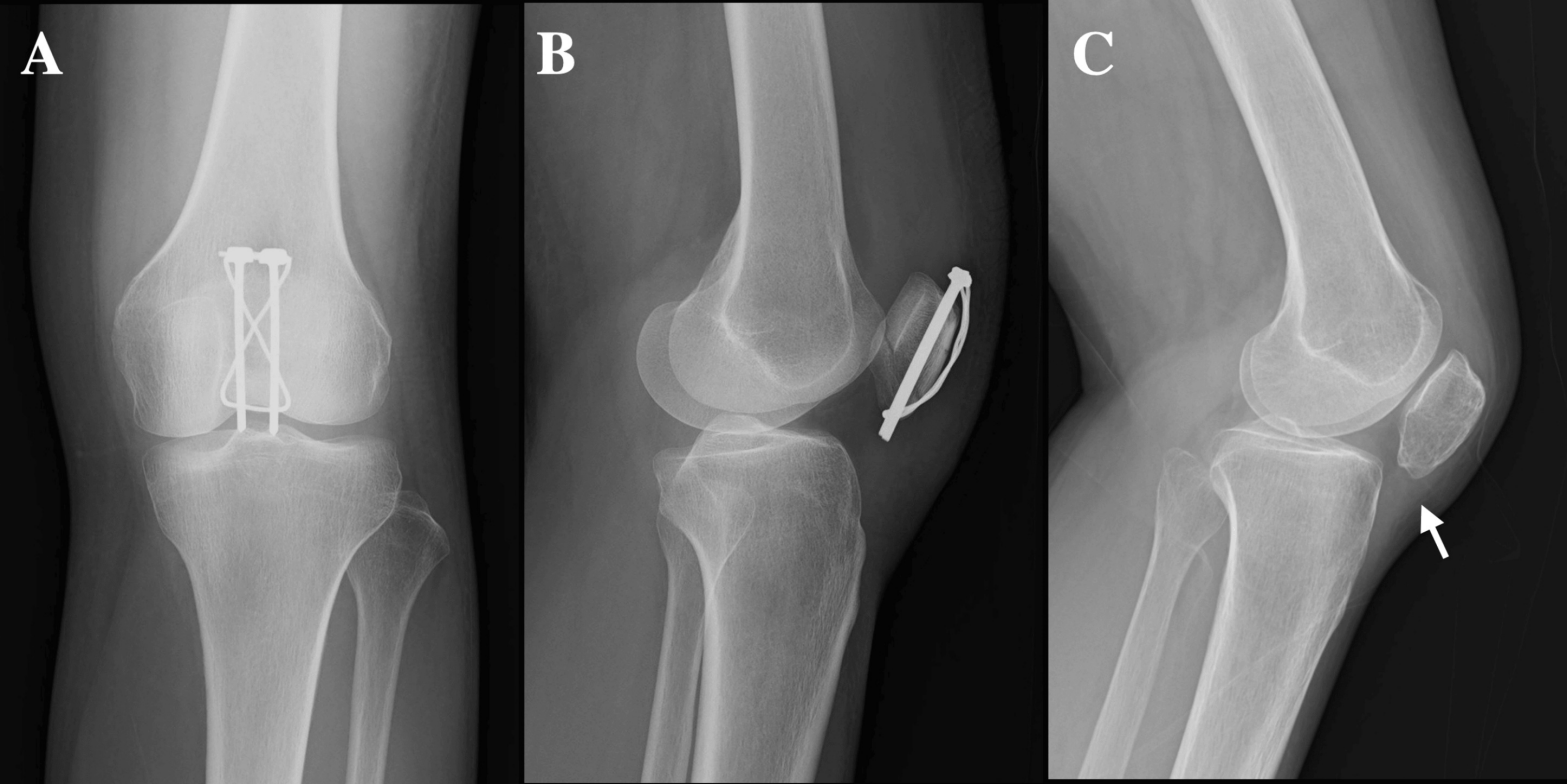
Preoperative radiographs Tension band wiring was performed for a patellar fracture (AO/OTA Type A1) at another institution (A, B). After implant removal, the patient was found to have marked patella baja (C, arrow).

A 59-year-old woman (height: 145 cm, weight: 42.5 kg, BMI: 20.21) sustained a patellar fracture (AO/OTA Type A1) due to trauma four years earlier and underwent tension band wiring at another institution (Figure 1A, 1B). After implant removal, she developed anterior knee pain during weight-bearing or knee-bending activities and was found to have marked patella baja (Figure 1C). Despite adequate rehabilitation, her symptoms persisted, and she was referred to our hospital one year after hardware removal.

Initial examination

At the initial presentation, there was no joint effusion or instability. The knee ROM was 0° extension and 120° flexion. Marked quadriceps atrophy was noted, with extension strength of 77 N on the affected side compared with 192.5 N on the contralateral side. Thigh circumference was 31.5 cm (contralateral: 33.0 cm). Visual Analogue Scale (VAS) scores were 36 mm (mild-moderate pain) during ambulation and 65 mm (moderate-severe pain) during knee flexion. The Timed Up and Go test (TUG) time was 12.89 seconds, with evident quadriceps weakness, gait disturbance, difficulty squatting, and inability to sit on the floor in the Japanese style (seiza).

Imaging findings

**Figure 2:**
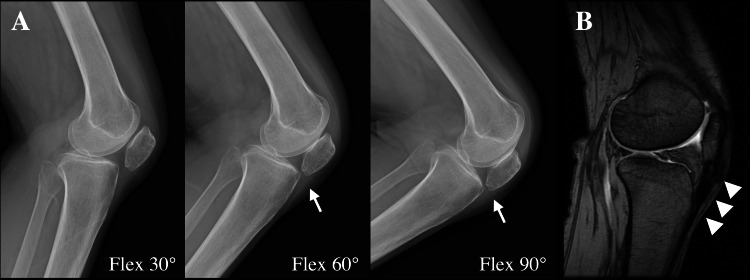
Preoperative imaging findings Lateral knee radiographs revealed progressive patella baja during flexion, with patellar impingement on the proximal tibia at 60° flexion (A, arrow). MRI showed a high-intensity signal in the proximal portion of a thickened patellar tendon (B, arrowhead).

Lateral knee radiographs revealed patellar deformity and progressive patella baja during flexion, with patellar impingement on the proximal tibia at 60° flexion (Figure 2A). MRI showed a high-intensity signal in the proximal portion of a thickened patellar tendon (Figure 2B). Patellar height was evaluated using the Insall-Salvati ratio (I/S ratio) [10] and Caton-Deschamps index (C/D index) [11]. At the time of fracture, the I/S ratio was 0.782 and the C/D index was 0.953, both within normal limits; however, at the initial visit, they had decreased markedly to 0.377 and 0.484, respectively (Table 1).

**Table 1:** Changes in patient condition, including patellar height indices, over time Patellar height was evaluated using the I/S ratio and C/D index, along with ROM, VAS at rest, and VAS during movement at each time point. C/D index, Caton-Deschamps index; I/S ratio, Insall-Salvati ratio; ROM, range of motion; VAS, Visual Analogue Scale

Time point	I/S ratio	C/D index	ROM	VAS at rest	VAS during movement
Patellar fracture	0.782	0.953	-	-	-
Wire removal	0.374	0.557	-	-	-
Pre-op	0.377	0.484	0-120°	36 mm	65 mm
Post-op, two years	0.935	1.169	0-150°	0 mm	25 mm
Healthy side	1.026	1.009	0-150°	0 mm	0 mm

Diagnosis

The patient was diagnosed with IPCS, in which pain and limited flexion were caused by patellotibial impingement secondary to patella baja. MRI findings suggested intratendinous signal changes without full-thickness disruption. Therefore, we planned proximalization of the tibial tubercle to avoid direct insult to the patellar tendon.

Surgical procedure

**Figure 3:**
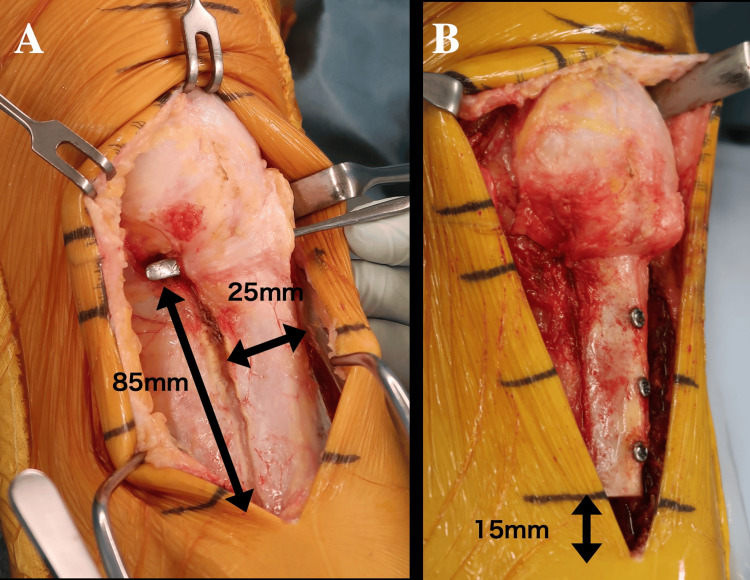
Intraoperative findings The medial and lateral joint capsules adjacent to the patellar tendon were incised to preserve its vascular supply (A). A V-shaped osteotomy of the tibial tubercle (85 × 25 × 10 mm) was performed. The tubercle was advanced proximally by 15 mm, and the fragment was secured using three 4-mm cannulated screws (B).

Diagnostic arthroscopy revealed no intra-articular abnormalities, including infrapatellar fat pad fibrosis. A 10-cm anterior incision was made over the proximal tibia, and the medial and lateral joint capsules adjacent to the patellar tendon were incised to preserve its vascular supply (Figure 3A). A V-shaped osteotomy of the tibial tubercle (85 × 25 × 10 mm) was performed. The proximal 15 mm of the osteotomized fragment was removed, and the tubercle was advanced proximally by 15 mm. Temporary fixation revealed improved flexion to 140°. The fragment was secured using three 4-mm cannulated screws, and the resected bone was grafted distally into the tibial defect (Figure 3B). The joint capsule and periosteum were closed, and the knee was immobilized in extension.

Postoperative course and rehabilitation

**Figure 4:**
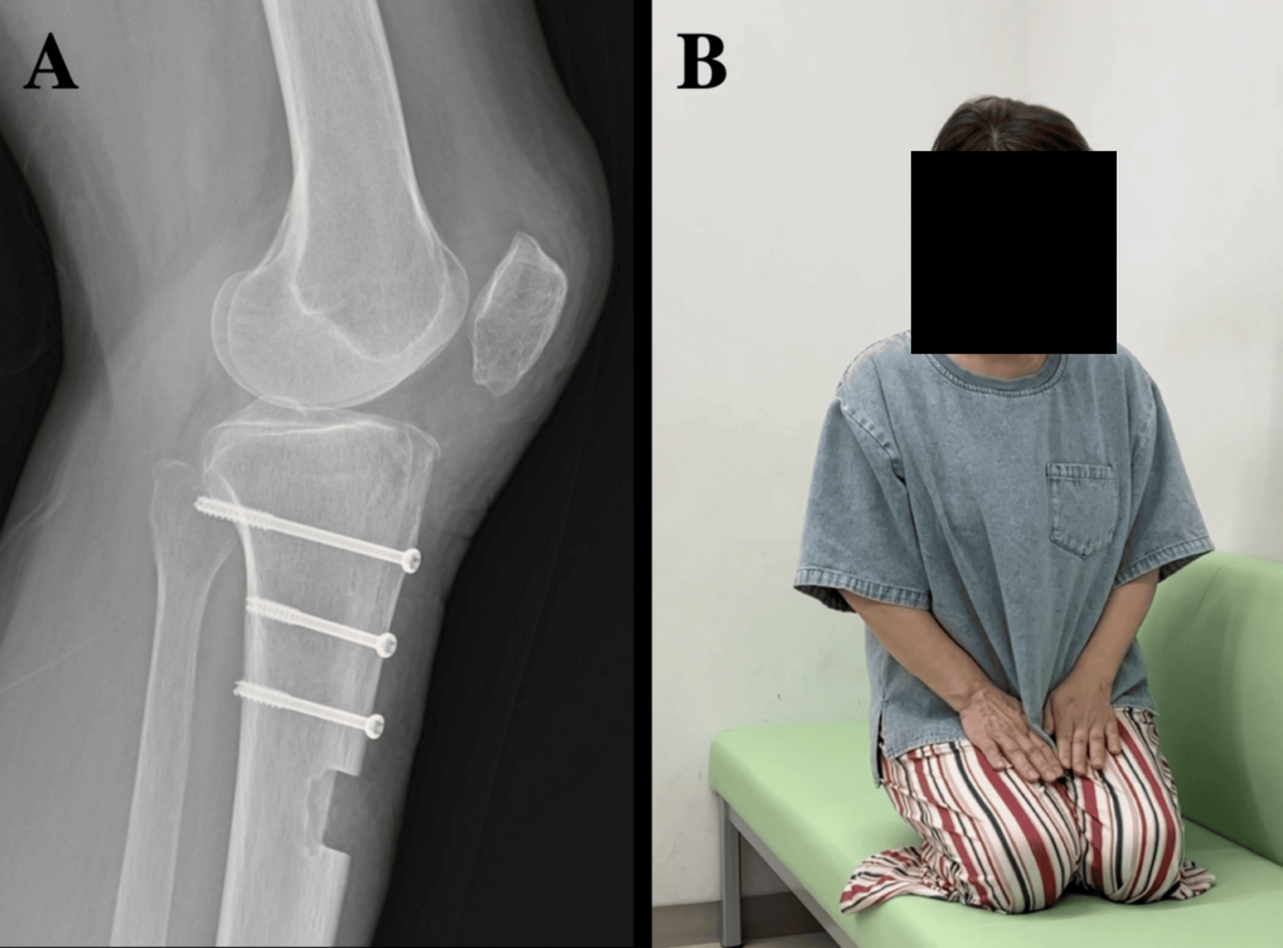
Changes in patellar height indices over time At one year, flexion reached 150°, with no evidence of patellar tendon shortening (A). Floor sitting in the Japanese style (seiza) was achieved (B).

Passive ROM (0-60°) was initiated on postoperative day one with an extension brace. Partial weight-bearing began at one week. ROM was limited to 0-90° for the first four weeks, after which full weight-bearing and unrestricted ROM were permitted. The brace was removed at four weeks. At three months, the extension lag had resolved, independent ambulation was possible, and flexion had improved to 135°. At one year, flexion reached 150°, with no evidence of patellar tendon shortening (Figure 4A). Hardware removal was performed because of screw irritation, and at the two-year follow-up, the patient showed symptom stabilization. Gait had normalized with resolution of limping, and full squatting and floor sitting in the Japanese style (seiza) were achieved (Figure 4B). Quadriceps strength was 86.4 N (contralateral: 96.6 N), thigh circumference was 33.0 cm bilaterally, VAS was 0 mm at rest and 25 mm during ambulation, and TUG had improved to 6.86 seconds.

## Discussion

IPCS is a rare but functionally debilitating condition that may develop following knee trauma or surgery. It is characterized by arthrofibrosis and mechanical impingement in the anterior knee, particularly affecting the patellofemoral joint [1]. Paulos et al. [2] described IPCS as a clinical entity with distinct stages, prodromal, active, and residual, each associated with progressive fibrosis, quadriceps atrophy, and restricted motion. However, there remains a lack of consensus regarding the diagnostic criteria and optimal treatment strategy for this condition.

In the present case, the patient developed IPCS following tension band wiring for a patellar fracture. After implant removal, persistent anterior knee pain, reduced flexion, and inability to perform daily activities such as squatting or floor sitting were noted. Radiographs and MRI demonstrated marked patella baja and patellotibial impingement, with significantly decreased patellar height indices (I/S ratio: 0.377; C/D index: 0.484). These findings, in conjunction with quadriceps atrophy and functional limitations, supported the diagnosis of residual-stage IPCS. Notably, MRI showed thickening and signal change in the patellar tendon without evidence of rupture, indicating preserved tendon continuity.

Various surgical options have been proposed for managing patella baja associated with IPCS. In et al. [3] reported patellar tendon lengthening using an Ilizarov external fixator, while Takai et al. [6] utilized a bone-patellar tendon-bone graft with anterior capsular advancement. Modified Z-plasty techniques for patellar tendon lengthening have also been described [7]. These procedures, while effective, often require direct manipulation or reconstruction of the patellar tendon, potentially increasing the risk of vascular compromise or further fibrosis.

In contrast, tibial tubercle proximalization offers a less invasive alternative by repositioning the insertion of the intact tendon, thereby restoring patellar height and relieving mechanical impingement. Drexler et al. [8] reported favorable outcomes following proximalization in patients with post-traumatic patella infera, with improvements in both patellar height and functional scores. While this technique has been widely employed for patellofemoral instability or cartilage lesions [12], recent studies have suggested its applicability in contracture-related disorders as well [9].

In our case, a 15-mm proximalization of the tibial tubercle led to immediate intraoperative improvement in knee flexion from 120° to 140°, with continued gains observed during follow-up. Several factors may have contributed to this favorable outcome: (1) preservation of the vascular supply of the patellar tendon by sparing the tendon itself and releasing only adjacent capsular structures; (2) rigid fixation of the osteotomized fragment, allowing early rehabilitation; and (3) a structured, stepwise rehabilitation program emphasizing gradual ROM restoration while minimizing stress on the extensor mechanism.

Patella baja occurs in approximately 25% of patients following surgical treatment for patellar fractures, with its incidence varying by fracture type [4]. AO/OTA classifications A1, C1.3, and C3 have been associated with a higher risk of developing this complication [4]. While patients with patella baja tend to exhibit reduced knee flexion compared with those without, no significant differences have been reported in long-term functional outcomes, such as the Böstman score [13] at two years postoperatively [4]. In the present case, the patient sustained a Type A1 fracture, which is known to carry an elevated risk for patella baja. However, surgical intervention for post-fracture patella baja remains rarely reported in the literature [3,6-8]. To our knowledge, few studies have addressed surgical correction in such cases, and outcomes remain poorly documented. This case highlights the need for further research and case accumulation to guide evidence-based management of patella baja following patellar fracture.

Compared with previously reported case series on IPCS and post-traumatic patella baja [3,6-9], the present case demonstrates several distinctive features. Most published series have described heterogeneous etiologies, such as post-total knee arthroplasty or multiligament injuries [3,6-9], and have predominantly employed surgical techniques involving direct manipulation or lengthening of the patellar tendon [3,6,7]. In contrast, our case involved a clearly defined etiology, post-fracture IPCS following an AO/OTA Type A1 patellar fracture, and was managed exclusively with tibial tubercle proximalization, thereby preserving tendon vascularity. Moreover, the functional assessment extended beyond conventional measures of ROM and pain scores, incorporating objective quadriceps strength testing, TUG performance, and culturally specific functional tasks such as squatting and floor sitting. Long-term follow-up over two years demonstrated sustained improvements in patellar height, muscle strength, and functional capacity.

An interesting finding in our case was the marked reduction in extension strength on the contralateral side, decreasing from 192.5 N to 96.6 N. We consider several possible explanations for this observation. First, prolonged disuse and altered gait mechanics before surgery may have led to bilateral quadriceps deconditioning, despite the absence of direct injury to the contralateral limb. Second, central neural adaptation and compensatory movement strategies during the period of limited mobility could have contributed to reduced voluntary activation of the quadriceps on the unaffected side, a phenomenon previously described in patients with unilateral knee pathology [14,15]. Finally, the patient’s postoperative rehabilitation program initially emphasized protection of the operated knee, which may have inadvertently limited use of the contralateral limb. This case, therefore, highlights the importance of incorporating bilateral lower limb strengthening into rehabilitation protocols, even when pathology appears to be unilateral.

Nonetheless, some limitations must be acknowledged. Despite the resolution of extension lag and improvement in flexion, the patient continued to experience some anterior knee discomfort during ambulation. This may reflect residual quadriceps weakness, persistent fibrosis, or irreversible changes to the extensor mechanism. Additionally, the long-term durability of the surgical correction remains uncertain, with potential risks including recurrence of baja, tendon elongation, or progressive anterior knee pain.

In conclusion, proximalization of the tibial tubercle may serve as a viable and effective surgical option for selected cases of IPCS, particularly those involving patella baja without full-thickness tendon disruption. This technique preserves the integrity of the patellar tendon, restores appropriate biomechanics, and allows for early rehabilitation. Further case accumulation and prospective long-term studies are warranted to validate the efficacy and safety of this approach in the management of IPCS.

## Conclusions

This case illustrates that tibial tubercle proximalization can offer meaningful clinical improvement in patients with IPCS associated with patella baja, particularly when conservative management has failed and the integrity of the patellar tendon is preserved. By elevating the tibial tubercle, patellar height can be restored without direct manipulation of the tendon itself, reducing the risk of further fibrosis or vascular compromise. The observed improvements in patellar height, knee flexion, quadriceps strength, and functional performance, including the ability to squat and sit in traditional positions, underscore the potential of this tendon-sparing technique. Moreover, the procedure allowed for a structured and progressive rehabilitation protocol, contributing to the overall functional recovery. Despite some residual anterior discomfort, the patient regained near-normal ROM and returned to daily activities with minimal limitations. This case adds to the growing body of evidence suggesting that tibial tubercle proximalization may be a practical and effective surgical strategy for selected IPCS cases. Continued evaluation of similar cases and long-term follow-up are essential to better define patient selection criteria, refine surgical techniques, and assess the durability of outcomes over time.

## References

[1] Paulos LE, Rosenberg TD, Drawbert J, Manning J, Abbott P (1987). Infrapatellar contracture syndrome: an unrecognized cause of knee stiffness with patella entrapment and patella infera. Am J Sports Med.

[2] Paulos LE, Wnorowski DC, Greenwald AE (1994). Infrapatellar contracture syndrome: diagnosis, treatment, and long-term followup. Am J Sports Med.

[3] In Y, Kim SJ, Kwon YJ (2007). Patellar tendon lengthening for patella infera using the Ilizarov technique. J Bone Joint Surg Br.

[4] Gao Y, Lin J, Hsu P, Wang Y, Zhu H, Wei H (2025). What factors are associated with patella baja after internal fixation of patellar fractures?. Clin Orthop Relat Res.

[5] Murase F, Takegami Y, Tokutake K (2025). Fracture of the patella involving inferior pole is associated with postoperative patella baja - a retrospective multicenter study. J Orthop Sci.

[6] Takai S, Shimazaki N, Yoshino N, Watanabe N, Nakachi N, Kobayashi M, Matsusita T (2009). New technique for knee flexion contracture with patella infera using patellar tendon reconstruction combined with anterior capsular shift: a case report. Arch Orthop Trauma Surg.

[7] Guido W, Christian H, Elmar H, Elisabeth A, Christian F (2016). Treatment of patella baja by a modified Z-plasty. Knee Surg Sports Traumatol Arthrosc.

[8] Drexler M, Dwyer T, Marmor M, Sternheim A, Cameron HU, Cameron JC (2013). The treatment of acquired patella baja with proximalize the tibial tuberosity. Knee Surg Sports Traumatol Arthrosc.

[9] Vandeputte FJ, Vandenneucker H (2017). Proximalisation of the tibial tubercle gives a good outcome in patients undergoing revision total knee arthroplasty who have pseudo patella baja. Bone Joint J.

[10] Insall J, Salvati E (1971). Patella position in the normal knee joint. Radiology.

[11] Caton J, Deschamps G, Chambat P, Lerat JL, Dejour H (1982). Patella infera. Apropos of 128 cases [Article in French]. Rev Chir Orthop Reparatrice Appar Mot.

[12] Tan SH, Ngiam EH, Lim JY, Lim AK, Hui JH (2021). Surgical management of patella alta in patellofemoral instability: a systematic review and meta-analysis. Orthop J Sports Med.

[13] Böstman O, Kiviluoto O, Nirhamo J (1981). Comminuted displaced fractures of the patella. Injury.

[14] Urbach D, Nebelung W, Weiler HT, Awiszus F (1999). Bilateral deficit of voluntary quadriceps muscle activation after unilateral ACL tear. Med Sci Sports Exerc.

[15] Christensen JC, Mizner RL, Foreman KB, Marcus RL, Pelt CE, LaStayo PC (2018). Quadriceps weakness preferentially predicts detrimental gait compensations among common impairments after total knee arthroplasty. J Orthop Res.

